# Impact of hypertension-related avoidable hospitalization on all-cause mortality in older patients with hypertension: a nationwide retrospective cohort study in Korea

**DOI:** 10.4178/epih.e2025019

**Published:** 2025-04-18

**Authors:** Yehrhee Son, Noorhee Son, Sungyoun Chun, Ki-Bong Yoo, Jung Hyun Chang, Woo-Ri Lee

**Affiliations:** 1 Division of Cancer Control and Policy, National Cancer Control Institute, National Cancer Center, Goyang, Korea; 2 Department of Research and Analysis, National Health Insurance Service Ilsan Hospital, Goyang, Korea; 3 Division of Health Administration, College of Software and Digital Healthcare Convergence, Yonsei University, Wonju, Korea; 4 Department of Otorhinolaryngology—Head and Neck Surgery, National Health Insurance Service Ilsan Hospital, Goyang, Korea

**Keywords:** Hypertension, Hospitalization, Death, Elderly, Health inequities

## Abstract

**OBJECTIVES:**

The prevalence of hypertension is increasing as a result of rapid population aging, which elevates the societal burden of the disease. In Korea, the hospitalization rate for hypertension-related admissions exceeds the average reported by the Organization for Economic Cooperation and Development; however, the impact of these hospitalizations has not been evaluated. Therefore, this study investigates the association between hypertension-related avoidable hospitalizations and all-cause mortality.

**METHODS:**

We included patients aged ≥60 years diagnosed with hypertension, identified using data from the National Health Insurance Services Senior Cohort spanning 2008 to 2019. The primary outcome was all-cause mortality measured at 3 years and 5 years after the hypertension diagnosis. The key independent variable was the incidence of hypertension-related avoidable hospitalizations within the first year following the initial hypertension diagnosis. Cox proportional hazards regression analysis was employed to assess these associations. To ensure robust findings and minimize selection bias, several sensitivity analyses were conducted.

**RESULTS:**

Out of 65,686 participants, 397 (0.6%) experienced hypertension-related avoidable hospitalizations within 1 year of their initial hypertension diagnosis. Individuals who experienced such hospitalizations had a significantly higher risk of all-cause mortality compared to those who did not (3-year: hazard ratio [HR], 2.12; 95% confidence interval [CI], 1.53 to 2.94; 5-year: HR, 2.13; 95% CI, 1.68 to 2.68).

**CONCLUSIONS:**

Hypertension-related avoidable hospitalizations among older adults are associated with an increased risk of both short-term and long-term all-cause mortality. These findings underscore the importance of timely hypertension management to prevent such hospitalizations.

## GRAPHICAL ABSTRACT

[Fig f3-epih-47-e2025019]

## Key Message

• Hypertension-related avoidable hospitalizations are significantly associated with both 3-year and 5-year all-cause mortality among older patients with hypertension in Korea.

• Socioeconomic and regional disparities were observed, with greater mortality risks among patients from low-income groups and non-metropolitan areas.

• Early and consistent hypertension management—including medication adherence—may help prevent avoidable hospitalizations and improve long-term survival outcomes.

## INTRODUCTION

Hypertension is defined as a systolic blood pressure (BP) of ≥140 mmHg or a diastolic BP of ≥90 mmHg [1]. Although often asymptomatic, hypertension can lead to severe complications over time—such as cardiovascular disease, ischemic heart disease, stroke, and chronic kidney disease—which significantly increase the risk of mortality; consequently, it is frequently referred to as a “silent killer” [2]. Older patients face particular challenges in managing hypertension because of factors including frailty, multimorbidity, and cognitive impairment [3]. As of 2019, approximately 1.3 billion people worldwide had hypertension [2], leading to 10.8 million deaths [4]. In 2021, hypertension was the leading risk factor for premature death and health deterioration globally among individuals aged ≥70 years [5]. In Korea, 12.3 million people had hypertension in 2021, with 43% of these cases observed in individuals aged 65 years or older [6]. The rising prevalence of hypertension—driven by rapid population aging—has increased the societal burden of the disease [6].

Outpatient care, including BP monitoring, medication adherence, and lifestyle modifications such as healthier dietary habits, can significantly reduce the risk of hypertension-related complications and mortality [1]. Therefore, hypertension is classified as an ambulatory care-sensitive condition (ACSC), because timely and consistent primary care can prevent hospital admissions [7-10]. Hospitalizations due to ACSCs are deemed avoidable since they can be prevented with adequate primary healthcare [11].

In Korea, enhancements in the diagnosis and treatment of hypertension have led to a decline in age-standardized and sex-standardized hospitalization rates for hypertension, from approximately 210 per 100,000 population in 2008 to 63 per 100,000 in 2021 [12]. Despite this progress, the 2021 hospitalization rate remains higher than the Organization for Economic Cooperation and Development (OECD) average of 52.8 per 100,000 [12]. Additionally, regional disparities in healthcare resources result in higher hypertension-related hospital admissions among rural older patients compared to those in urban areas, highlighting significant regional health inequities [13].

Previous studies have concentrated on identifying risk factors for hypertension-related avoidable hospitalizations [14-19]. However, the short-term and long-term impacts of these avoidable hospitalizations on patient outcomes, including mortality, have not been thoroughly investigated. This study aimed to examine the association between hypertension-related avoidable hospitalizations and all-cause mortality.

## MATERIALS AND METHODS

### Data and study population

In Korea, the National Health Insurance Service (NHIS) operates as a single-payer system and has provided universal health coverage since 1989 [20]. Approximately 97% of the population is covered by NHIS health insurance, while the remaining 3% are supported by a Medical Aid program [21]. Korean citizens pay monthly health insurance premiums based on their income and assets; in return, the NHIS covers most healthcare costs, excluding out-of-pocket expenses [22,23]. Because healthcare providers bill the NHIS directly for services, the NHIS maintains comprehensive healthcare utilization data for the entire population. Consequently, NHIS data are considered nationally representative.

**Figure 1.**
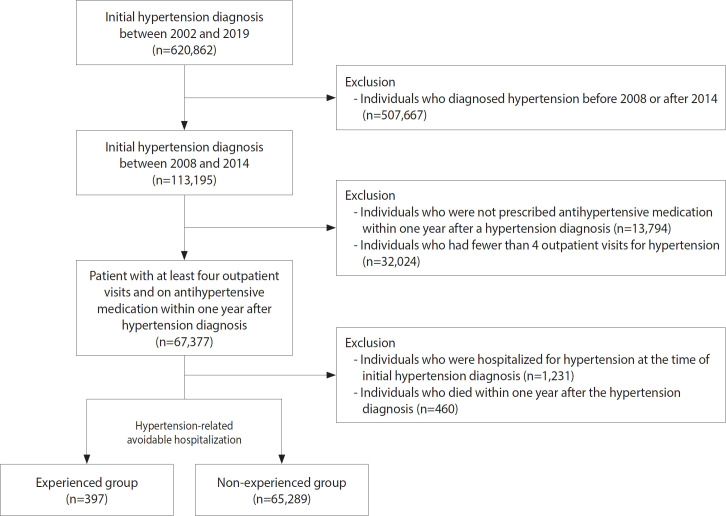
Data flow chart.

This study utilized data from the NHIS-Senior Cohort covering the years 2008 to 2019. The NHIS-Senior Cohort is a stratified random sample representing approximately 8% of older adults aged 60-80 years in 2008 and was further categorized by age, sex, health insurance premiums, and region [24]. The study population comprised older patients aged ≥60 years who were first diagnosed with hypertension (International Classification of Diseases, 10th revision [ICD-10] code: I10) as the principal diagnosis between 2008 and 2014 (n=113,195). Recognizing that sole reliance on ICD-10 codes might include non-hypertensive patients due to inherent discrepancies in claims data, we further restricted inclusion to individuals who were prescribed antihypertensive medications within 1 year of their initial hypertension diagnosis (n=99,401) and who had at least 4 outpatient visits (n=67,377). The list of antihypertensive drugs used to define the inclusion criteria is presented in Supplementary Material 1. Individuals hospitalized for hypertension at the time of initial diagnosis (n=1,231) and those who died within 1 year of diagnosis (n=460) were excluded. The final study sample comprised 65,686 older patients with hypertension (Figure 1).

### Variables

#### Outcome measures

The primary outcome was all-cause mortality. To assess the impact of the independent variable, both short-term and long-term effects were evaluated. The short-term effect was defined as all-cause mortality occurring within 3 years of the hypertension diagnosis, while the long-term effect was defined as all-cause mortality within 5 years of diagnosis.

#### Independent variable

Avoidable hospitalizations are commonly used as indicators of primary care accessibility and quality, with their occurrence suggesting potential shortcomings in 1 or both areas [25]. The variable of interest in this study was hypertension-related avoidable hospitalizations. The OECD defines primary hypertension-related avoidable hospitalizations as hospital admissions for hypertension classified under the ICD-10 code I10 [26]. In accordance with this OECD definition, hypertension-related avoidable hospitalizations were defined as admissions with a primary diagnosis of “I10, I119, I129, I139” within 1 year of the initial hypertension diagnosis. Only the first occurrence of a hypertension-related avoidable hospitalization was considered; recurrent events were excluded from the analysis.

#### Covariates

The covariates in this study were categorized into socioeconomic, health-related, and hypertension-related factors. Socioeconomic factors included sex (male or female), age (60s, 70s, or ≥80s), income (below or above median income), region (metropolitan or non-metropolitan), and type of healthcare insurance (Medical Aid, National Health Insurance [NHI] for the self-employed, or NHI for employees). Health-related factors included disability status (non-disabled or disabled) and the Charlson comorbidity index (CCI) score (0, 1, 2, or ≥3). Hypertension-related factors comprised the medication possession ratio (MPR), ischemic heart disease, cerebrovascular disease, and the year of hypertension diagnosis. The MPR, an indicator of medication adherence, was used to evaluate the extent to which patients adhered to prescribed antihypertensive medications [27]. Patients with an MPR of ≥80% were classified as adherent, whereas those with an MPR of <80% were classified as non-adherent [27]. The MPR was calculated as follows:


MPR (%)=(Number of days prescribed antihypertensive medication within 365 days after initial hypertension diagnosis/365 days)×100

Ischemic heart disease and cerebrovascular disease were included owing to their close association with hypertension and were used to adjust for disease severity. Ischemic heart disease was identified using ICD-10 codes I20-I25, and cerebrovascular disease was defined using ICD-10 codes I60-I69 as the principal diagnosis at the time of hypertension diagnosis.

### Statistical analysis

The analyses in this study were conducted in 3 stages. First, a chi-squared test was employed to evaluate the relationship between the general characteristics of the study population and both short-term and long-term all-cause mortality, with a p-value threshold of <0.05 indicating statistical significance. Second, survival analysis was performed to examine the association between hypertension-related avoidable hospitalizations and both short-term and long-term all-cause mortality. The proportional hazards assumption was verified using Kaplan-Meier survival curves, and the statistical significance of these curves was assessed with the log-rank test (p<0.05). Cox proportional hazards models were then used for regression analysis, with statistical significance determined when the 95% confidence interval (CI) did not include 1. All survival analyses—including Kaplan-Meier curves and number-at-risk tables—were based on the time to the first occurrence of a hypertension-related avoidable hospitalization; recurrent events were not considered. Third, sensitivity analyses were conducted using 2 approaches—subgroup analysis and propensity score matching (PSM)—to reinforce the robustness of our findings and reduce selection bias. First, PSM, a quasi-experimental method that approximates randomized clinical trials in observational studies, was applied to ensure greater comparability between case and control groups [28]. We employed the greedy matching method with a 1:3 ratio, and the adequacy of matching was assessed using the standardized mean difference, with an absolute threshold of <0.1. Following PSM, a Cox regression analysis was conducted on the matched cohort. Second, subgroup analyses were performed to explore potential disparities in the association between hypertension-related avoidable hospitalizations and all-cause mortality across different socioeconomic and regional subgroups. Additionally, a subgroup analysis based on MPR adherence was conducted to evaluate the effect of proper hypertension management. Prior to these analyses, a joint test was used to assess the interaction effect with hypertension-related avoidable hospitalization, with statistical significance defined at a p-value threshold of <0.05. All regression analyses were adjusted for the covariates included in the study. Given that hypertension-related avoidable hospitalizations are time-dependent variables that may recur, the landmark method—a robust approach for controlling immortal time bias—was applied [29]. Because the outcome measure was all-cause mortality, patients experiencing hypertension-related avoidable hospitalizations inherently had an immortal time period from the time of hypertension diagnosis until the hospitalization event, during which death could not occur. To address this bias, a landmark period of 1 year from the date of hypertension diagnosis was established. Under this method, only the exposure status within the landmark period was considered, and any exposures occurring afterward were disregarded [29]. Consequently, the observation window for hypertension-related avoidable hospitalizations was defined as the 1-year landmark period, and individuals who experienced the outcome event during this period were excluded. All analyses were performed using SAS version 9.4 (SAS Institute Inc., Cary, NC, USA) and R version 4.3.0 (R Foundation for Statistical Computing, Vienna, Austria).

### Ethics statement

The Institutional Review Board of the National Health Insurance Service Ilsan Hospital, Korea waived ethical approval for this study (NHIMC 2023-06-022) because only secondary data containing anonymized and encrypted personal information were used.

## RESULTS

The relationship between the general characteristics of the study population and all-cause mortality is presented in Table 1. Among the total study population of 65,686 individuals, 397 (0.6%) experienced hypertension-related avoidable hospitalizations within 1 year of their initial hypertension diagnosis. Regarding 3-year all-cause mortality, 37 participants (9.3%) who experienced hypertension-related avoidable hospitalizations died, compared to 1,912 participants (2.9%) who did not, indicating a significantly higher mortality rate in those with such hospitalizations (p<0.001). Similarly, for 5-year all-cause mortality, 73 participants (18.6%) with hypertension-related avoidable hospitalizations died compared to 4,154 participants (6.4%) without, further demonstrating a higher mortality rate among those with these hospitalizations (p<0.001).

**Figure 2.**
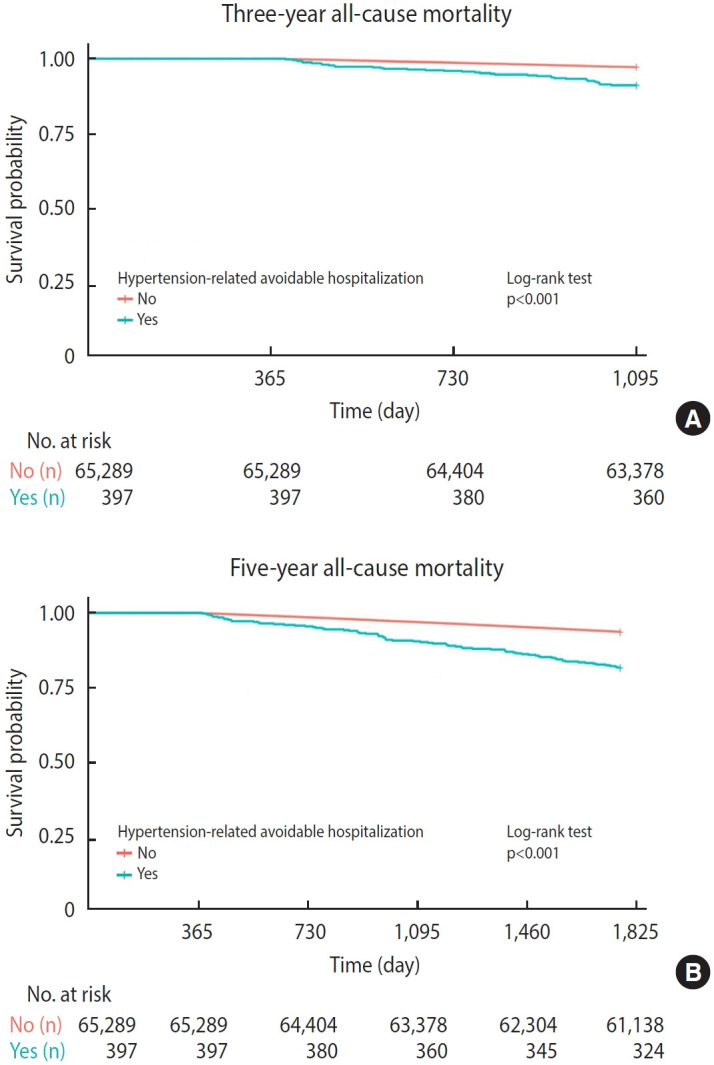
Kaplan-Meier survival curve.

The results of the Cox regression analysis examining the association between hypertension-related avoidable hospitalizations and short-term and long-term all-cause mortality are presented in Table 2. The proportional hazards assumption was verified using Kaplan-Meier survival curves, which revealed no violations. The survival curves for different hospitalization statuses were statistically significantly different (Figure 2; p<0.001). According to the Cox regression analysis, individuals who experienced hypertension-related avoidable hospitalizations had a higher risk of all-cause mortality compared to those who did not (3-year: HR, 2.12; 95% CI, 1.53 to 2.94; 5-year: HR, 2.13; 95% CI, 1.68 to 2.68).

The results of the sensitivity analysis, which was conducted to reinforce the robustness of our findings and reduce selection bias, are presented in Table 3. The PSM results indicated that all standardized mean differences were below the absolute threshold of 0.1, confirming adequate matching (Supplementary Material 2). The Cox regression analysis on the matched cohort demonstrated that individuals with hypertension-related avoidable hospitalizations had a significantly higher risk of all-cause mortality compared to those without (3-year: HR, 2.10; 95% CI, 1.37 to 3.20; 5-year: HR, 2.19; 95% CI, 1.62 to 2.95). In addition to the PSM analysis, subgroup analyses were performed to explore potential disparities in the association between hypertension-related avoidable hospitalizations and all-cause mortality across different socioeconomic and regional subgroups; these results are also presented in Table 3. The joint test for the interaction between income group and hypertension-related avoidable hospitalization was statistically significant for both short-term and long-term all-cause mortality (p<0.001). Notably, individuals in the below-median income group who experienced such hospitalizations had a higher risk of both short-term and long-term all-cause mortality (3-year: HR, 2.47; 95% CI, 1.66 to 3.70; 5-year: HR, 2.48; 95% CI, 1.87 to 3.31). Among those in the above-median income group, the 5-year all-cause mortality risk was higher for individuals with hypertension-related avoidable hospitalizations (HR, 1.69; 95% CI, 1.13 to 2.53). The joint test for the interaction between regional group and hypertension-related avoidable hospitalization was also statistically significant for both short-term and long-term mortality (p<0.001). Individuals in the metropolitan group who experienced such hospitalizations had a higher risk of both short-term and long-term all-cause mortality (3-year: HR, 2.11; 95% CI, 1.19 to 3.76; 5-year: HR, 1.99; 95% CI, 1.30 to 3.05). Similarly, individuals in the non-metropolitan group with these hospitalizations had higher risks (3-year: HR, 2.11; 95% CI, 1.42 to 3.15; 5-year: HR, 2.16; 95% CI, 1.64 to 2.86). The joint test for the interaction between MPR group and hypertension-related avoidable hospitalization was statistically significant only for long-term all-cause mortality (p=0.003). Within the non-adherence group, individuals with these hospitalizations had a higher risk of long-term mortality (5-year: HR, 1.60; 95% CI, 1.07 to 2.38), while in the adherence group, those experiencing such hospitalizations had a higher risk of both short-term and long-term mortality (3-year: HR, 2.85; 95% CI, 1.93 to 4.21; 5-year: HR, 2.55; 95% CI, 1.91 to 3.39).

## DISCUSSION

### Key findings

This study investigated the association between hypertension-related avoidable hospitalizations and both short-term and long-term all-cause mortality in older patients with hypertension. The analysis revealed that older patients who experienced such hospitalizations had an increased risk of mortality in both the short-term and long-term. Moreover, this association was particularly pronounced among individuals with below-median incomes and those living in non-metropolitan areas.

### Interpretation

The study’s findings indicate that hypertension-related avoidable hospitalizations elevate the risk of mortality, which aligns with previous research on the link between avoidable hospitalizations and mortality [30,31]. Since these hospitalizations adversely affect both short-term and long-term health outcomes, timely management of hypertension is essential for preventing hospitalization [32]. The study focused on hospitalizations occurring within the first year after the initial hypertension diagnosis. Despite this focus on the early post-diagnosis period, the results suggest that such events considerably influence later health outcomes, emphasizing the need for proactive management from the time of diagnosis. Previous research has demonstrated that early management of hypertension can lead to improved health outcomes [33-35]. Consistent with these findings, our results showed that patients in the adherence group (MPR ≥80%) during the first year after diagnosis experienced better short-term and long-term outcomes than those in the non-adherence group (MPR <80%). Additionally, we observed that the adherence group had a lower long-term incidence of hypertension-related avoidable hospitalizations compared to the non-adherence group (Supplementary Material 3). Korea’s primary care hypertension guidelines underscore the importance of improving the MPR to prevent health deterioration in patients with hypertension [36]. However, according to the Korean Hypertension Fact Sheet [6], the BP control rate among adults aged 65 and older (defined as systolic BP<140 mmHg and diastolic BP<90 mmHg) was only 59.4% between 2019 and 2021, indicating that nearly one-third of older hypertensive patients struggle to achieve adequate control. These findings highlight the need for targeted education to encourage active health management and appropriate treatment from the early stages of diagnosis.

This study also identified socioeconomic disparities in the relationship between hypertension-related avoidable hospitalizations and mortality. The results are consistent with previous studies that have highlighted income-related and region-related health disparities among patients with hypertension [13,37]. High-income patients typically have greater awareness of the importance of hypertension prevention and management, as well as better access to treatment [37]. In contrast, low-income patients exhibit lower health literacy [38], engage in unhealthy behaviors, and have a higher likelihood of comorbidities [39]. To reduce health disparities among older patients with hypertension, comprehensive BP management strategies and active education on healthy lifestyles are necessary. Regionally, Korea experiences significant differences in healthcare infrastructure between metropolitan and rural areas [13]. These infrastructural differences are closely linked to healthcare accessibility and contribute to regional disparities [40]. For example, data published by Korea’s National Medical Center in 2022 showed that nearly 90% of Seoul residents could access emergency care within 30 minutes, compared to only 44% and 41% of residents in rural areas such as Gangwon and Gyeongsang Provinces, respectively [41]. Similarly, 2020 data from the Ministry of Land, Infrastructure, and Transport revealed that the average distance to primary care facilities was 0.97 km in Seoul versus 11.05 km in Gangwon Province [42]. In our study, we found a higher incidence of hypertension-related avoidable hospitalizations among individuals living in non-metropolitan areas (Supplementary Material 4). These region-based findings suggest that higher hospitalization rates may reflect poorer primary care quality or limited healthcare accessibility [25]. Thus, the observed disparities could be attributed to differences in either the quality of or access to primary care across regions. However, since we did not include direct measures of primary care quality or accessibility, this interpretation remains indirect. Future studies should incorporate explicit indicators of primary care quality and accessibility to validate these findings further.

### Limitations

This study had several limitations. First, it did not account for health behaviors such as smoking status, alcohol consumption, and physical activity, all of which may affect patients’ baseline health conditions. To partly address this limitation, adjustments were made for health-related and hypertension-related factors at the time of diagnosis. Second, given the nature of claims data, discrepancies may exist between recorded diagnostic codes and the actual diagnoses made by healthcare providers. To minimize such discrepancies, we included only patients who had at least 4 outpatient visits with hypertension as the primary diagnosis and who were prescribed antihypertensive medications, thereby reducing potential errors related to diagnostic code mismatches. Third, the time-dependent nature of the variable of interest raises the possibility of immortal time bias. We addressed this bias by employing the landmark method, an effective technique for its control. Fourth, complications arising during the follow-up period were not captured. Patients who experience a hypertension-related avoidable hospitalization are at higher risk for subsequent complications, particularly cardiovascular and cerebrovascular diseases, which may affect mortality. Although baseline cardiovascular and cerebrovascular diseases were adjusted for in our analysis, future studies should include new complications over a longer follow-up period. Fifth, patients hospitalized for hypertension-related avoidable events may have had a greater disease burden, reflecting more severe hypertension than those not hospitalized. To mitigate this, we adjusted for the CCI, ischemic heart disease, and cerebrovascular disease, and we conducted a sensitivity analysis using PSM to reduce selection bias. Despite these measures, residual confounding related to disease severity may persist.

Despite these limitations, this study is significant because it identifies a clear association between hypertension-related avoidable hospitalization and adverse short-term and long-term health outcomes in older patients with hypertension.

In conclusion, hypertension-related avoidable hospitalizations in older patients were associated with an increased risk of both short-term and long-term all-cause mortality. These findings underscore the importance of reducing such hospitalizations through timely and effective hypertension management. Although avoidable hospitalizations are generally considered indicators of primary care quality or limited healthcare accessibility, our study did not directly assess these factors. Therefore, any conclusions regarding primary care quality or accessibility should be made with caution. Nonetheless, our results suggest that enhancing primary care quality and improving healthcare accessibility may help reduce avoidable hospitalizations and ultimately improve outcomes in older adults with hypertension. Future research should incorporate explicit measures of primary care access, quality, and continuity to further validate these associations.

## Figures and Tables

**Figure f3-epih-47-e2025019:**
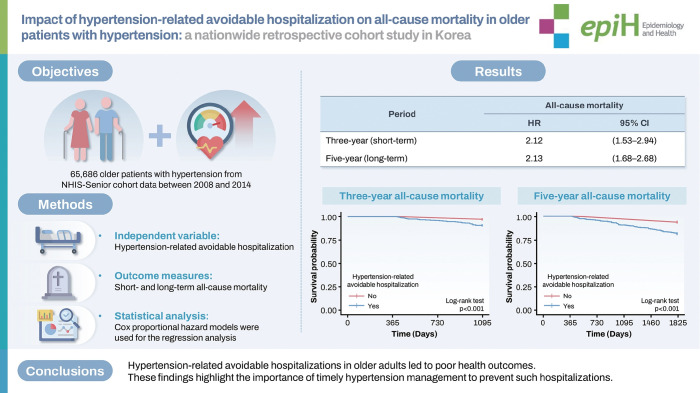


**Table 1.** General characteristics of the study population

Characteristics	All-cause mortality					
	Three-year			Five-year		
	Yes	No	p-value	Yes	No	p-value
Total	1,949 (3.0)	63,737 (97.0)		4,227 (6.4)	61,459 (93.6)	
Hypertension-related avoidable hospitalization			<0.001			<0.001
No	1,912 (2.9)	63,377 (97.1)		4,154 (6.4)	61,135 (93.6)	
Yes	37 (9.3)	360 (90.7)		73 (18.6)	324 (81.6)	
MPR (%)			<0.001			<0.001
Non-adherence (<80)	721 (3.8)	18,253 (96.2)		1,475 (7.8)	17,499 (92.2)	
Adherence (≥80)	1,228 (2.6)	45,484 (97.4)		2,752 (5.9)	43,960 (94.1)	
Sex			<0.001			<0.001
Male	1,136 (4.0)	27,394 (96.0)		2,431 (8.5)	26,099 (91.5)	
Female	813 (2.2)	36,343 (97.8)		1,796 (4.8)	35,360 (95.2)	
Age			<0.001			<0.001
60s	613 (1.6)	38,468 (98.4)		1,300 (3.3)	37,781 (96.7)	
70s	1,012 (4.3)	22,420 (95.7)		2,241 (9.6)	21,191 (90.4)	
≥80	324 (10.2)	2,849 (89.8)		686 (21.6)	2,487 (78.4)	
Income			0.003			<0.001
Lower income group	1,057 (3.2)	32,387 (96.8)		2,315 (6.9)	31,129 (93.1)	
Higher income group	892 (2.8)	31,350 (97.2)		1,912 (5.9)	30,330 (94.1)	
Region			0.002			<0.001
Metropolitan	747 (2.7)	26,625 (97.3)		1,604 (5.9)	25,768 (94.1)	
Non-metropolitan	1,202 (3.1)	37,112 (96.9)		2,623 (6.8)	35,691 (93.2)	
Type of health insurance			<0.001			<0.001
Medical aid	275 (5.8)	4,429 (94.2)		596 (12.7)	4,108 (87.3)	
NHI self employed	598 (3.0)	19,624 (97.0)		1,293 (6.4)	18,929 (93.6)	
NHI employee	1,076 (2.6)	39,684 (97.4)		2,338 (5.7)	38,422 (94.3)	
Disability			<0.001			<0.001
Non-disabled	1,493 (2.6)	55,665 (97.4)		3,306 (5.8)	53,852 (94.2)	
Disabled	456 (5.3)	8,072 (94.7)		921 (10.8)	7,607 (89.2)	
CCI						<0.001
0	644 (2.1)	30,353 (97.9)	<0.001	1,498 (4.8)	29,499 (95.2)	
1	342 (2.7)	12,409 (97.3)		834 (6.5)	11,917 (93.5)	
2	359 (3.0)	11,696 (97.0)		792 (6.6)	11,263 (93.4)	
≥3	604 (6.1)	9,279 (93.9)		1,103 (11.2)	8,780 (88.8)	
Ischemic heart disease			0.008			<0.001
No	1,776 (2.9)	59,092 (97.1)		3,820 (6.3)	57,048 (93.7)	
Yes	173 (3.6)	4,645 (96.4)		407 (8.4)	4,411 (91.6)	
Cerebrovascular disease			<0.001			<0.001
No	1,741 (2.8)	59,858 (97.2)		3,769 (6.1)	57,830 (93.9)	
Yes	208 (5.1)	3,879 (94.9)		458 (11.2)	3,629 (88.8)	
Year of hypertension diagnosis			0.311			0.234
2008	376 (3.1)	11,775 (96.9)		839 (6.9)	11,312 (93.1)	
2009	324 (2.9)	10,705 (97.1)		706 (6.4)	10,323 (93.6)	
2010	294 (3.0)	9,622 (97.0)		636 (6.4)	9,280 (93.6)	
2011	303 (3.2)	9,066 (96.8)		607 (6.5)	8,762 (93.5)	
2012	273 (3.0)	8,887 (97.0)		585 (6.4)	8,575 (93.6)	
2013	199 (2.6)	7,436 (97.4)		454 (5.9)	7,181 (94.1)	
2014	180 (2.8)	6,246 (97.2)		400 (6.2)	6,026 (93.8)	

Values are presented as number (%). MPR, medication possession ratio; NHI, National Health Insurance; CCI, Charlson comorbidity index.

**Table 2.** Results of the multivariable Cox regression analysis of the association between hypertension-related avoidable hospitalization and all-cause mortality

Variables	All-cause mortality	
	Three-year	Five-year
Hypertension-related avoidable hospitalization		
No	1.00 (reference)	1.00 (reference)
Yes	2.12 (1.53, 2.94)	2.13 (1.68, 2.68)
MPR (%)		
Non-adherence (<80)	1.36 (1.23, 1.49)	1.24 (1.16, 1.32)
Adherence (≥80)	1.00 (reference)	1.00 (reference)
Sex		
Male	2.18 (1.99, 2.39)	2.19 (2.06, 2.33)
Female	1.00 (reference)	1.00 (reference)
Age		
60s	1.00 (reference)	1.00 (reference)
70s	2.77 (2.50, 3.07)	3.00 (2.79, 3.21)
≥80	7.06 (6.15, 8.12)	7.64 (6.94, 8.40)
Income		
Lower income group	1.09 (0.99, 1.20)	1.12 (1.05, 1.20)
Higher income group	1.00 (reference)	1.00 (reference)
Region		
Metropolitan	1.00 (reference)	1.00 (reference)
Non-metropolitan	1.01 (0.92, 1.11)	1.03 (0.97, 1.10)
Type of health insurance		
Medical aid	1.46 (1.26, 1.69)	1.53 (1.39, 1.69)
NHI self employed	1.16 (1.05, 1.28)	1.15 (1.07, 1.23)
NHI employee	1.00 (reference)	1.00 (reference)
Disability		
Non-disabled	1.00 (reference)	1.00 (reference)
Disabled	1.59 (1.43, 1.77)	1.49 (1.39, 1.61)
CCI		
0	1.00 (reference)	1.00 (reference)
1	1.18 (1.40, 1.35)	1.24 (1.14, 1.35)
2	1.40 (1.23, 1.60)	1.33 (1.22, 1.45)
≥3	2.49 (2.22, 2.79)	1.97 (1.82, 2.14)
Ischemic heart disease		
No	1.00 (reference)	1.00 (reference)
Yes	0.96 (0.82, 1.13)	1.09 (0.98, 1.21)
Cerebrovascular disease		
No	1.00 (reference)	1.00 (reference)
Yes	1.31 (1.13, 1.52)	1.37 (1.24, 1.52)
Year of hypertension diagnosis		
2008	1.45 (1.20, 1.74)	1.35 (1.19, 1.53)
2009	1.38 (1.14, 1.66)	1.27 (1.12, 1.44)
2010	1.36 (1.12, 1.64)	1.26 (1.10, 1.43)
2011	1.42 (1.18, 1.72)	1.22 (1.07, 1.39)
2012	1.23 (1.01, 1.49)	1.14 (0.99, 1.29)
2013	1.09 (0.89, 1.34)	1.06 (0.93, 1.22)
2014	1.00 (reference)	1.00 (reference)

Values are presented as hazard ratio (95% confidence interval). MPR, medication possession ratio; NHI, National Health Insurance; CCI, Charlson comorbidity index.

**Table 3.** Results of the sensitivity analysis

Sensitivity analysis		All-cause mortality			
		Three-year	p-value^1^	Five-year	p-value^1^
Propensity score matching					
Hypertension-related avoidable hospitalization					
No		1.00 (reference)		1.00 (reference)	
Yes		2.10 (1.37, 3.20)		2.19 (1.62, 2.95)	
Subgroup analysis					
Income					
Lower income	Hypertension-related avoidable hospitalization		<0.001		<0.001
	No	1.00 (reference)		1.00 (reference)	
	Yes	2.47 (1.66, 3.70)		2.48 (1.87, 3.31)	
Higher income	Hypertension-related avoidable hospitalization				
	No	1.00 (reference)		1.00 (reference)	
	Yes	1.72 (0.97, 3.04)		1.69 (1.13, 2.53)	
Region					
Metropolitan	Hypertension-related avoidable hospitalization		<0.001		<0.001
	No	1.00 (reference)		1.00 (reference)	
	Yes	2.11 (1.19, 3.76)		1.99 (1.30, 3.05)	
Non-metropolitan	Hypertension-related avoidable hospitalization				
	No	1.00 (reference)		1.00 (reference)	
	Yes	2.11 (1.42, 3.15)		2.16 (1.64, 2.86)	
MPR (%)					
Non-adherence (<80)	Hypertension-related avoidable hospitalization		0.139		0.003
	No	1.00 (reference)		1.00 (reference)	
	Yes	1.35 (0.74, 2.46)		1.60 (1.07, 2.38)	
Adherence (≥80)	Hypertension-related avoidable hospitalization				
	No	1.00 (reference)		1.00 (reference)	
	Yes	2.85 (1.93, 4.21)		2.55 (1.91, 3.39)	

Values are presented as hazard ratio (95% confidence interval). MPR: medication possession ratio.

1 Using joint test.

## Supplementary materials

Supplementary Material 1. List of active pharmaceutical ingredient codes for antihypertensive medications

Supplementary Material 2. General characteristics of the study population before and after propensity score matching

Supplementary Material 3. Hypertension-related avoidable hospitalization survival curve by medication possession ratio

Supplementary Material 4. Hypertension-related avoidable hospitalization survival curve by residential area
