# Supplementary material for: Impact of hypertension-related avoidable hospitalization on all-cause mortality in older patients with hypertension: a nationwide retrospective cohort study in Korea
Source: Epidemiol Health. 2025 Apr 18;47:e2025019. doi: 10.4178/epih.e2025019 (PMC12178768; doi:10.4178/epih.e2025019)
Supplement: Supplementary Material 1. — List of active pharmaceutical ingredient codes for antihypertensive medications [file epih-47-e2025019-Supplementary-1.docx]

**Supplementary Material 1.** List of active pharmaceutical ingredient codes for antihypertensive medications

| **Active pharmaceutical ingredient** | | |
| --- | --- | --- |
| **Major category** | **Middle category** | **Code** |
| β-blocker | Arotinolol | 110201ATB, 110202ATB |
|  | Atenolol | 111402ATB, 111403ATB, 262100ATB, 483101ATB, 483102ATB |
|  | Betaxolol | 116830COS, 116831COS, 116801ATB, 116803ATB |
|  | Bevantolol | 117001ATB, 117002ATB |
|  | Bisoprolol | 117903ATB, 117904ATB, 469800ATB, 469900ATB, 470000ATB, 117901ATB, 117902ATB |
|  | Carteolol | 124830COS, 124832COS, 124833COS, 124801ATB |
|  | Carvedilol | 125001ATB, 125002ATB, 125003ATB, 125004ACR, 125005ATB, 125006ACR, 125007ACR, 125007ATR, 125008ACR, 125008ATR |
|  | Celiprolol | 129101ATB |
|  | Labetalol | 180230BIJ, 180231BIJ |
|  | Metoprolol | 262400ATR, 193802ATB, 194003ATR |
|  | Nadolol | 198301ATB |
|  | Nebivolol | 489501ATB, 489502ATB, 489503ATB, 683000ATB, 683100ATB, 683200ATB, 691200ATB, 693000ATB |
|  | Propranolol | 219901ATB, 219904ATB |
| α-blockers | Doxazosin | 149102ATB, 149104ATR |
|  | Terazosin | 235501ATB, 235502ATB, 235503ATB |
| Angiotensin-converting enzyme inhibitor | Alacepril | 104201ATB, 104202ATB |
|  | Captopril | 122901ATB, 122902ATB, 122903ATB |
|  | Cilazapril | 133001ATB, 133002ATB, 133003ATB |
|  | Enalapril | 151601ATB, 151603ATB |
|  | Lisinopril | 184501ATB |
|  | Perindopril | 211301ATB, 211302ATB, 501601ATB, 501602ATB, 556200ATB |
|  | Ramipril | 222401ATB, 222402ATB, 222404ATB, 447100ATB, 447200ATB |
|  | Zofenopril | 510401ATB, 510402ATB, 510403ATB |
| Angiotensin II receptor blocker | Azilsartan | 662401ATB, 662402ATB, 662403ATB, 673500ATB, 673600ATB |
|  | Candesartan | 122601ATB, 122602ATB, 122603ATB, 423700ATB, 652900ATB, 653000ATB, 653100ATB, 661800ATB, 661900ATB, 662000ATB, 662100ATB, 673700ATB |
|  | Eprosartan | 429201ATB, 460500ATB |
|  | Fimasartan | 515201ATB, 515202ATB, 515203ATB, 522000ATB, 526800ATB, 651900ATB, 652000ATB, 652100ATB, 652700ATB, 654700ATB, 654800ATB, 654900ATB, 655000ATB, 684300ATB, 684400ATB, 684500ATB, 684600ATB, 684700ATB, 688100ATB, 688200ATB, 688300ATB, 688400ATB, 688500ATB |
|  | Irbesartan | 177301ATB, 177303ATB, 385700ATB, 385800ATB, 524000ATB, 524100ATB, 527000ATB, 527100ATB |
|  | Losartan | 185701ATB, 185702ATB, 185703ATB, 262500ATB, 378900ATB, 486900ATB, 502700ATB, 503000ATB, 513900ATB, 637400ATB, 637500ATB, 637600ATB, 662800ATB, 662900ATB, 663000ATB, 663900ATB, 664000ATB, 664100ATB, 664200ATB, 664300ATB, 664400ATB, 692000ATB, 692100ATB, 692200ATB, 692300ATB, 692400ATB, 692500ATB |
|  | Olmesartan | 468501ATB, 468502ATB, 468503ATB, 500500ATB, 582200ATB, 582400ATB, 513600ATB, 519700ATB, 519800ATB, 520000ATB, 547600ATB, 547700ATB, 547800ATB, 547900ATB, 548000ATB, 631300ATB, 629400ATB, 629500ATB, 629600ATB, 632800ATB, 632900ATB, 633000ATB, 526300ATB, 526400ATB, 526500ATB, 526900ATB, 644100ATB, 644200ATB, 653200ATB, 677300ATB, 677400ATB, 677500ATB, 686800ATB, 686900ATB |
|  | Telmisartan | 378801ATB, 378802ATB, 378803ATB, 443200ATB, 443300ATB, 502600ATB, 511500ATB, 511600ATB, 511700ATB, 623100ATB, 521200ATB, 521300ATB, 521400ATB, 644800ATB, 663500ATB, 663600ATB, 663700ATB, 663800ATB, 682700ATB, 682800ATB, 682900ATB, 697300ATB, 697400ATB, 697500ATB, 697600ATB, 629900ATB, 630000ATB, 630100ATB, 630200ATB, 631600ATB, 631700ATB, 671200ATB, 671300ATB, 671400ATB, 671500ATB, 671600ATB, 671700ATB, 677000ATB, 677100ATB |
|  | Valsartan | 247101ATB, 247102ATB, 247103ATB, 247104ATB, 356400ATB, 442600ATB, 492800ATB, 492900ATB, 495800ATB, 522200ATB, 522300ATB, 522400ATB, 522600ATB, 522700ATB, 522800ATB, 522900ATB, 523000ATB, 523100ATB, 523200ATB, 523300ATB, 523400ATB, 525000ATB, 525100ATB, 525200ATB, 525300ATB, 629700ATB, 629800ATB, 634900ATB, 635000ATB, 635100ATB, 635200ATB, 651401ATB, 651402ATB, 651403ATB, 679500ATB, 679600ATB, 679700ATB, 680300ATB, 691400ATB, 691500ATB, 690400ATB, 690500ATB, 690600ATB, 690700ATB |
| Vasodilator | Hydralazine | 170701ATB, 170730BIJ |
|  | Minoxidil | 196102ATB |
|  | Nitroprusside | 229330BIJ |
| Calcium channel blocker | Amlodipine | 107601ATB, 107602ATB, 107603ATB, 459801ACH, 459801ATB, 459802ACH, 459901ATB, 459902ATB, 464601ATB, 470801ATB, 470802ATB, 476201ATB, 479701ATB, 483201ATB, 483202ATB, 486501ATB, 486502ATB, 486503ATB, 492800ATB, 492900ATB, 495800ATB, 500500ATB, 582200ATB, 582400ATB, 502700ATB, 503000ATB, 513900ATB, 511500ATB, 511600ATB, 511700ATB, 623100ATB, 519700ATB, 519800ATB, 520000ATB, 521200ATB, 521300ATB, 521400ATB, 644800ATB, 522600ATB, 522700ATB, 522800ATB, 522900ATB, 523000ATB, 523100ATB, 523200ATB, 523300ATB, 523400ATB, 547600ATB, 547700ATB, 547800ATB, 547900ATB, 548000ATB, 631300ATB, 629400ATB, 629500ATB, 629600ATB, 632800ATB, 632900ATB, 633000ATB, 637400ATB, 637500ATB, 637600ATB, 651900ATB, 652000ATB, 652100ATB, 652700ATB, 652900ATB, 653000ATB, 653100ATB, 662800ATB, 662900ATB, 663000ATB, 663500ATB, 663600ATB, 663700ATB, 663800ATB, 682700ATB, 682800ATB, 682900ATB, 697300ATB, 697400ATB, 697500ATB, 697600ATB, 472300ATB, 472400ATB, 472500ATB, 518900ATB, 614500ATB, 663900ATB, 664000ATB, 664100ATB, 664200ATB, 664300ATB, 664400ATB, 671200ATB, 671300ATB, 671400ATB, 671500ATB, 671600ATB, 671700ATB, 677000ATB, 677100ATB, 673900ATB, 674000ATB, 674100ATB, 678600ATB, 677300ATB, 677400ATB, 677500ATB, 686800ATB, 686900ATB, 679500ATB, 679600ATB, 679700ATB, 680300ATB, 691400ATB, 691500ATB, 684300ATB, 684400ATB, 684500ATB, 684600ATB, 684700ATB, 690400ATB, 690500ATB, 690600ATB, 690700ATB, 692000ATB, 692100ATB, 692200ATB, 692300ATB, 692400ATB, 692500ATB |
|  | Barnidipine | 114001ACH, 114002ACH, 114003ACH |
|  | Benidipine | 115101ATB, 115102ATB, 115103ATB, 115104ATB |
|  | Cilnidipine | 133101ATB, 133102ATB |
|  | Diltiazem | 145703ACR, 145706ATB, 145707ACR, 145707ATR, 145704BIJ |
|  | Efonidipine | 441201ATB, 441202ATB |
|  | Felodipine | 157501ATR, 157503ATR, 262400ATR, 447100ATB, 447200ATB |
|  | Lacidipine | 180301ATB, 180302ATB, 180303ATB |
|  | Lercanidipine | 182001ATB, 522200ATB, 522300ATB, 522400ATB |
|  | Manidipine | 188001ATB, 188002ATB |
|  | Nicardipine | 201003ACR, 201030BIJ, 201031BIJ, 201033BIJ |
|  | Nifedipine | 201405ATR, 201409ATR, 528201ATR |
|  | Nisoldipine | 356202ATR, 356203ATR |
|  | Verapamil | 247603ATR, 247605ATR, 247606ATB, 247607ATB, 247630BIJ |
