# Supplementary material for: Impact of hypertension-related avoidable hospitalization on all-cause mortality in older patients with hypertension: a nationwide retrospective cohort study in Korea
Source: Epidemiol Health. 2025 Apr 18;47:e2025019. doi: 10.4178/epih.e2025019 (PMC12178768; doi:10.4178/epih.e2025019)
Supplement: Supplementary Material 2. — General characteristics of the study population before and after propensity score matching [file epih-47-e2025019-Supplementary-2.docx]

| **Supplementary Material 2.** General characteristics of the study population before and after propensity score matching | | | | | | | | | |
| --- | --- | --- | --- | --- | --- | --- | --- | --- | --- |
| **Variable** | **Before PSM** | | | | **After PSM** | | | | |
|  | **Hypertensive avoidable hospitalization** | | | | **Hypertensive avoidable hospitalization** | | | | |
|  | **No** | | **Yes** | | **No** | | **Yes** | | **Standardized  mean  difference** |
|  | **N** | **%** | **No** | **%** | **N** | **%** | **No** | **%** |  |
| **Total** | 65,289 | (99.4) | 397 | (0.6) | 1,184 | (74.9) | 396 | (25.1) |  |
| **MPR** |  |  |  |  |  |  |  |  |  |
| Non-adherence (＜80%) | 18,839 | (99.3) | 135 | (0.7) | 397 | (74.6) | 135 | (25.4) |  |
| Adherence (≥80%) | 46,450 | (99.4) | 262 | (0.6) | 787 | (75.1) | 261 | (24.9) | 0.01209 |
| **Sex** |  |  |  |  |  |  |  |  |  |
| Male | 28,389 | (99.5) | 141 | (0.5) | 407 | (74.3) | 141 | (25.7) |  |
| Female | 36,900 | (99.3) | 256 | (0.7) | 777 | (75.3) | 255 | (24.7) | 0.02527 |
| **Age** |  |  |  |  |  |  |  |  |  |
| 60s | 38,888 | (99.5) | 193 | (0.5) | 559 | (74.3) | 193 | (25.7) |  |
| 70s | 23,278 | (99.3) | 154 | (0.7) | 470 | (75.4) | 153 | (24.6) | 0.02193 |
| Over 80s | 3,123 | (98.4) | 50 | (1.6) | 155 | (75.6) | 50 | (24.4) | 0.01667 |
| **Income** |  |  |  |  |  |  |  |  |  |
| Below income group | 33,228 | (99.4) | 216 | (0.6) | 671 | (75.7) | 215 | (24.3) |  |
| Above income group | 32,061 | (99.4) | 181 | (0.6) | 513 | (73.9) | 181 | (26.1) | -0.04768 |
| **Region** |  |  |  |  |  |  |  |  |  |
| Metropolitan | 27,235 | (99.5) | 137 | (0.5) | 422 | (75.5) | 137 | (24.5) |  |
| Other cities | 38,054 | (99.3) | 260 | (0.7) | 762 | (74.6) | 259 | (25.4) | -0.02160 |
| **Type of healthcare insurance** |  |  |  |  |  |  |  |  |  |
| Medical Aid | 4,645 | (98.7) | 59 | (1.3) | 207 | (78.1) | 58 | (21.9) |  |
| NHI Self employed | 20,119 | (99.5) | 103 | (0.5) | 299 | (74.4) | 103 | (25.6) | -0.01681 |
| NHI Employee | 40,525 | (99.4) | 235 | (0.6) | 678 | (74.3) | 235 | (25.7) | -0.04259 |
| **Disability** |  |  |  |  |  |  |  |  |  |
| Non-disabled | 56,838 | (99.4) | 320 | (0.6) | 954 | (74.9) | 320 | (25.1) |  |
| Disabled | 8,451 | (99.1) | 77 | (0.9) | 230 | (75.2) | 76 | (24.8) | 0.00637 |
| **CCI** |  |  |  |  |  |  |  |  |  |
| 0 | 30,894 | (99.7) | 103 | (0.3) | 310 | (75.1) | 103 | (24.9) |  |
| 1 | 12,657 | (99.3) | 94 | (0.7) | 288 | (75.4) | 94 | (24.6) | 0.01430 |
| 2 | 11,966 | (99.3) | 89 | (0.7) | 259 | (74.4) | 89 | (25.6) | -0.01491 |
| ≥3 | 9,772 | (98.9) | 111 | (1.1) | 327 | (74.8) | 110 | (25.2) | -0.00394 |
| **Ischemic heart disease** |  |  |  |  |  |  |  |  |  |
| No | 60,539 | (99.5) | 329 | (0.5) | 1,020 | (75.6) | 329 | (24.4) |  |
| Yes | 4,750 | (98.6) | 68 | (1.4) | 164 | (71.0) | 67 | (29.0) | -0.09481 |
| **Cerebrovascular disease** |  |  |  |  |  |  |  |  |  |
| No | 61,252 | (99.4) | 347 | (0.6) | 1,046 | (75.1) | 347 | (24.9) |  |
| Yes | 4,037 | (98.8) | 50 | (1.2) | 138 | (73.8) | 49 | (26.2) | -0.02478 |
| **Diagnosed year of hypertension** |  |  |  |  |  |  |  |  |  |
| 2008 | 12,064 | (99.3) | 87 | (0.7) | 256 | (74.9) | 86 | (25.1) | 0.00083 |
| 2009 | 10,954 | (99.3) | 75 | (0.7) | 239 | (76.1) | 75 | (23.9) |  |
| 2010 | 9,848 | (99.3) | 68 | (0.7) | 188 | (73.4) | 68 | (26.6) |  |
| 2011 | 9,308 | (99.3) | 61 | (0.7) | 170 | (73.6) | 61 | (26.4) |  |
| 2012 | 9,114 | (99.5) | 46 | (0.5) | 147 | (76.2) | 46 | (23.8) |  |
| 2013 | 7,600 | (99.5) | 35 | (0.5) | 119 | (77.3) | 35 | (22.7) |  |
| 2014 | 6,401 | (99.6) | 25 | (0.4) | 65 | (72.2) | 25 | (27.8) |  |
| ** PSM: Propensity score matching; MPR: Medication possession ratio; CCI: Charlson comorbidity index* | | | | | | | | | |
