# Supplementary material for: Impact of hypertension-related avoidable hospitalization on all-cause mortality in older patients with hypertension: a nationwide retrospective cohort study in Korea
Source: Epidemiol Health. 2025 Apr 18;47:e2025019. doi: 10.4178/epih.e2025019 (PMC12178768; doi:10.4178/epih.e2025019)

**Supplementary Material 3.** Hypertension-related avoidable hospitalization survival curve by medication possession ratio


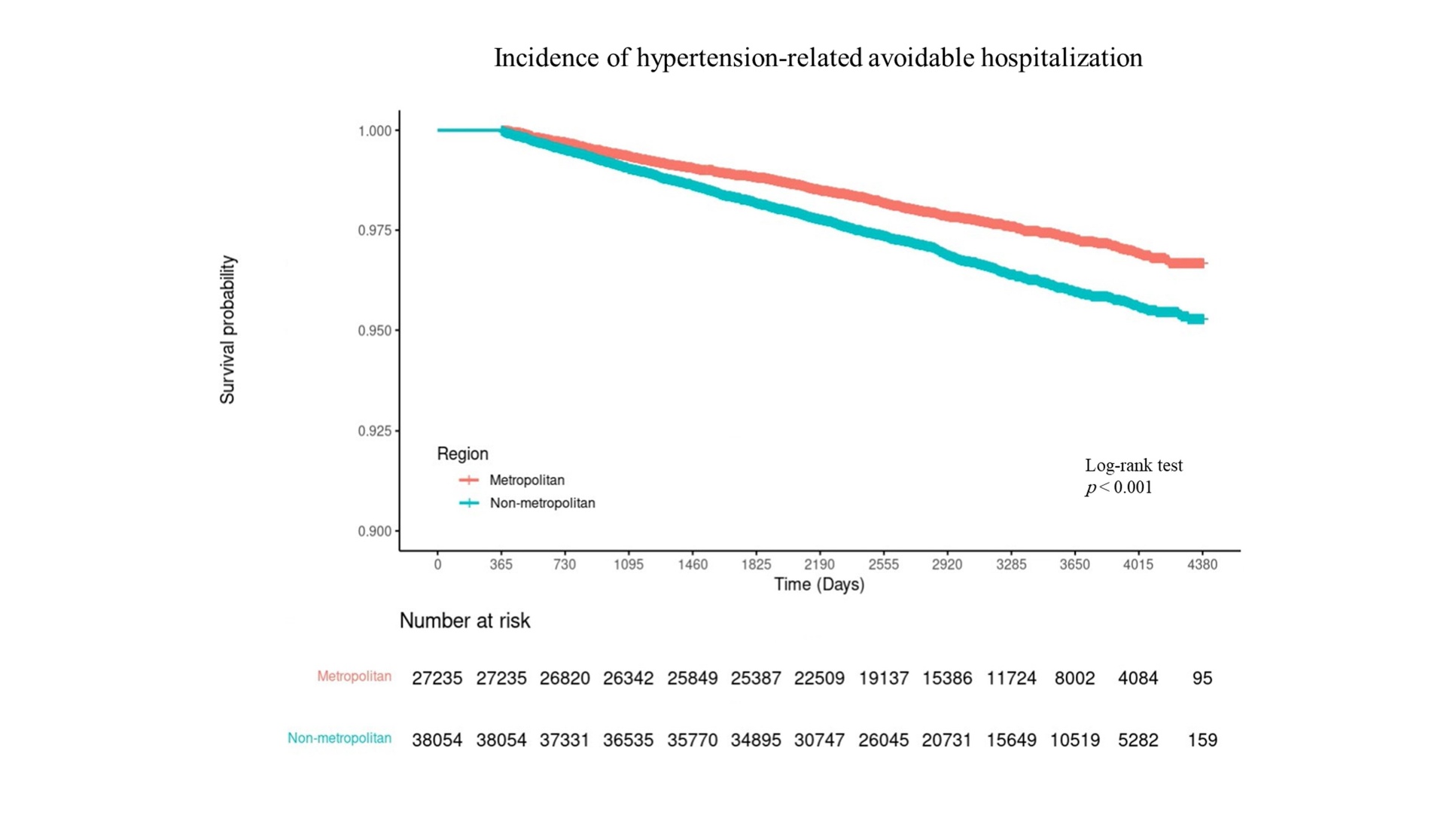

Supplement: Supplementary Material 3. — Hypertension-related avoidable hospitalization survival curve by medication possession ratio [file epih-47-e2025019-Supplementary-3.docx]
